# Coerced addiction treatment: Client perspectives and the implications of their neglect

**DOI:** 10.1186/1477-7517-7-13

**Published:** 2010-06-20

**Authors:** Karen A Urbanoski

**Affiliations:** 1 Centre for Addiction and Mental Health, 33 Russell St. Toronto, ON, M5S 2S1, Canada

## Abstract

Recent work has criticized the evidence base for the effectiveness of addiction treatment under social controls and coercion, suggesting that the development of sound policies and treatment practices has been hampered by numerous limitations of the research conducted to date. Implicit assumptions of the effectiveness of coerced treatment are evident in the organization and evolution of treatment, legal, and social service systems, as well as in related legislative practices. This review builds upon previous work by focusing in greater detail on the potential value of incorporating client perspectives on coercion and the implications for interpreting and applying existing research findings. Reviewing the existing empirical and theoretical literature, a case is made for greater accuracy in representing coercive experiences and events in research, so as to better align the measured concepts with actual processes of treatment entry and admission. Attention is given to studies of the effectiveness of treatment under social controls or pressures, the connections to coercion and decision-making, and theoretical perspectives on motivation and behaviour change, including Self-Determination Theory in particular. This synthesis of the available research on coerced addiction treatment suggests that it remains largely unclear to what extent many of the commonly employed methods for getting people into treatment may be detrimental to the treatment process and longer-term outcomes. The impact of coercion upon individual clients, treatment systems, and population health has not been adequately dealt with by addiction researchers to date.

## Review

In a recent review, Wild outlined a comprehensive criticism of the evidence base for the effectiveness of treatment under social controls and coercion, suggesting that the development of sound policies and treatment practices has been hampered by numerous limitations of the research conducted to date [1]. As limitations, he highlighted the focus on non-empirical arguments defending or denouncing the use of coercive strategies; the prioritization of legal strategies over other forms of pressure; lack of recognition of the heterogeneity in the implementation of coercive strategies; the neglect of potential iatrogenic effects to individuals, programs, and the system as a whole; and exclusion of stakeholder (i.e., client and service provider) perspectives on coercion. These arguments lay in distinct contrast to an apparently growing consensus that "coercion works" and is a viable strategy for promoting treatment participation [2-4].

This review builds upon the previous work of Wild and other researchers [1,5-7], focusing in greater detail on the potential value of incorporating client perspectives on coercion and considering the implications of their neglect for interpreting and applying existing research findings. It is argued that the evidence base supporting coerced addiction treatment remains weak, and that much could be gained by a revision of the coercion construct, including both expansion of its purview and better specification of the domains involved. Reviewing the existing empirical and theoretical literature, a case is made for greater accuracy in representing coercive experiences and events in research, so as to better align the measured concepts with actual processes of treatment entry and admission. Using a more thoughtful approach toward studying coercion, a more meaningful and consistent set of findings may arise than what has been possible to glean from research to date.

Questions of the effectiveness of using coercive strategies to promote, encourage or force people to enter addiction treatment are highly relevant to present-day treatment systems. Implicit assumptions of the effectiveness of treatment under such circumstances are evident in the organization and evolution of treatment, legal, and social service systems, as well as in related legislative practices. In 2006 in the US, a system-wide estimate of 38% of admissions to publicly-funded addiction treatment programs were referred by the criminal justice system [8]. In 2008-09 in Ontario, Canada's most populous province, 22% of those in specialized, public treatment reported a condition attached to treatment entry, including treatment as a condition of probation or parole, child custody, receipt of social assistance, continued school attendance or family contact [9]. Just over 8% reported being referred by the legal system, with a similar proportion referred by friends or family.

Formalized mechanisms for treatment referral and collaboration with the legal system continue to evolve and expand, exemplified by mandatory programs for license reinstatement following convictions for impaired driving and an ever-expanding litany of drug treatment and other problem-solving courts. Growth of workplace alcohol and drug treatment programs contributed to system expansion throughout the 1980s [10], while the role of substance abuse as a barrier to employment and economic self-sufficiency figured largely into debates over welfare reform in North America in the 1990s [11-13]. Within the past decade in Canada, legislation and policies have allowed for the expansion of drug treatment courts across the country [14], as well as for a civil commitment approach towards treatment for adolescents in the province of Alberta [15] and making financial benefits contingent upon treatment participation for some recipients of social assistance in Ontario [16].

Against this backdrop, the sections that follow provide a critical review of the existing body of empirical and theoretical work on coercion, with attention to the conceptual shortcomings of typical definitions and study designs. Implications for future research design and measurement, and for local treatment practices and system policies, are considered.

### Search strategy

This review involved a search of the English-language academic and evaluation literature pertaining to social pressures and coercion to enter addiction treatment. Electronic databases, including PsycInfo, Pubmed/Medline, and the Campbell and Cochrane Collaborations, were searched for the following keywords: substance abuse treatment; perceived coercion; mandates or pressure; compulsory or forced treatment; motivation, readiness to change, or treatment readiness; self-determination or autonomy; and outcomes. Additional searches were made of publication and library catalogues of the author's home institutions (the Centre for Addiction and Mental Health and the University of Toronto) and other substance policy and research organizations, including the Canadian Centre on Substance Abuse, Substance Abuse and Mental Health Services Administration, National Institutes for Health, and the European Monitoring Centre on Drugs and Drug Addiction. Only original empirical and review articles were reviewed, excluding anecdotal and opinion pieces. Studies focusing on the use of coercive strategies *throughout *treatment as opposed to at treatment admission were also excluded, as they are out of the scope of the present review. This includes studies of the effectiveness of applying sanctions and rewards throughout treatment, and the impact of legal or other third-party monitoring and surveillance of the treatment process. Although the research strategy was thorough, the focus on English-language literature places some restrictions on the generalizability of the content. Specifically, the majority of studies were conducted in the US, with smaller numbers from Canada, Australia and the UK.

### Social controls versus coercion: drawing the distinction

As noted by Wild [1], most research to date has defined coerced treatment in terms of referral source or the presence of monitoring conditions or reinforcements, neglecting the implications for client motivation, interest, or intent in pursuing treatment. Research has also largely restricted its focus to pressures or mandates administered by legal authorities, downplaying those mitigated by other social agents [1]. However, a variety of non-legal governmental and other institutions also play an important role in encouraging or mandating treatment entry, as do informal social networks.

Wild provides a helpful distinction between coerced treatment and treatment under social controls [1]. The term *coercion *is reserved to describe situations in which clients perceive a lack of control over the decision to enter treatment. In other words, *coerced treatment *refers to that which is perceived as an imposition and an infringement on autonomy, regardless of the agent or source. This is distinguished from treatment under *social controls*, which refer to the wide range of mandates and pressures that are objectively applied to ensure or encourage treatment entry, but do not explicit account for client perceptions or assigned meanings. These have been classified broadly in terms of their source. *Legal *pressures include civil commitment, court-ordered treatment, and diversion-to-treatment programs, such as drug treatment courts. *Formal non-legal *pressures are those mitigated by non-legal institutions or systems, including mandatory treatment referrals by employers, schools, children's aid or social assistance programs. *Informal *social pressures refer to forms of interpersonal persuasion, including threats and ultimatums by friends and family. Similar distinctions between coercion and mandates or pressures have been recommended elsewhere [17-19].

Both conceptually and empirically, the constructs of social controls and coercion are related. Pressures and mandates from legal, other formal, and informal sources have all been linked with greater perceived coercion [20]. Similarly, legal problems and mandates from legal authorities, employers, and social services are associated with lower autonomous motivation and higher controlled motivation for treatment [21]. The role of informal social network pressures in experiences of coercion is more complex. While informal pressures to quit or reduce substance use and/or to enter treatment are associated with greater controlled motivation [21,22], social network opposition to continued substance use has also been associated with greater autonomous motivation for abstinence [23]. Acting in accordance with the norms and expectations of one's social network may support motivational processes in other ways (e.g., via supporting a need for social relatedness), such that clients may not necessarily experience pressures from this source as threatening to their autonomy.

Despite these associations, there is ample empirical evidence supporting the lack of a direct, or one-to-one, correspondence between objective pressure strategies and perceptions of coercion [1]. Legal mandates are not uniformly reported as coercive, or even necessarily perceived as a deciding factor in entering treatment [24-26]. Similarly, treatment that is not legally mandated appears to nonetheless often involve the avoidance of negative external contingencies on the part of clients [27]. In a study of clients entering outpatient counselling, one-third of those who were legally mandated and two-thirds of employer mandated clients reported no coercion to enter treatment, while over one-third of self-referred clients reported at least some coercion [20]. In a multivariable analysis of predictors of client interest in and initial level of commitment to treatment, internal motivation, but not the objective presence of pressures from legal, other formal, and informal sources, predicted more positive attitudes toward treatment [21]. That is, the measure reflecting the underlying reasons for seeking treatment was a relatively more important predictor of orientation toward treatment than were the objective measures of social events.

These findings highlight that it can not be assumed that external pressures are always tied to the decision to enter addiction treatment [28,29]. People seeking treatment are often experiencing multiple internal and external pressures [27,30-32], and the importance of any given one is likely dependent on a host of individual and contextual factors. In addition, many individuals with substance problems initially seek help from services outside the addiction treatment sector, depending on how they and those around them define their problems [33]. In the same way, it is equally problematic to equate self-referrals with voluntarism in seeking treatment. Self-referred clients may nonetheless be avoiding legal or employer sanctions and still perceive a great deal of coercion to enter treatment.

Theoretical work on self-determination further supports the idea that it is perceptions of coercion and threats to autonomy, rather than their objective presence *per se*, that have implications for motivation and behaviour change. Among social-psychological models for studying health behaviour change, Self-Determination Theory (SDT) is unique in its consideration of autonomy as the central concept [34-36]. It provides a useful framework for studies of coerced treatment by addressing how social events are perceived and how those perceptions affect motivation and behaviour [1]. SDT also distinguishes between autonomous and controlled forms of motivation, based on the reasons for initiating behaviour, its realized adaptive value, and degree of environmental support, among other factors. Briefly, activities enacted out of a sense of personal need and value are autonomously motivated, while those enacted because of external pressures and demands are considered controlled or externally motivated. By linking the degree to which behaviours are integrated and internally valued to their persistence and effectiveness and, in turn, to psychological well-being, SDT provides a rich set of hypotheses concerning the role of autonomy in mechanisms of treatment-assisted recovery. Importantly, by explicitly allowing for differences in the ways that people respond to external events and social contexts, it highlights the inadequacy of considering only external circumstances when addressing coercion.

The distinction between autonomous and controlled forms of motivation represents the major distinguishing factor of SDT from other theories of motivation and behaviour change, such as the stages of change or Transtheoretical Model (TTM) [37]. Differences in the behaviour change process hypothesized by SDT versus TTM stem mainly from the ways that motivation is formed and expected to change over time. The stages of change construct does not account for why some people undertake behaviour change while others do not, which is problematic from the perspective of evaluating the relative effectiveness of social control strategies or coercion to initiate the behaviour change process. An individual who engages in an activity because of perceptions that it is required by others would not be differentiated in level of motivation from another who engages in an activity out of a sense of personal commitment. However, personal valuation of treatment and recovery is possibly an important determinant of positive outcomes in the long-term [34].

### Effectiveness of treatment under social controls and coercion

Although conflicting and negative findings are reported with respect to the effectiveness of treatment under legal pressures or mandates [6,7,38], studies have largely found that that legal pressures promote longer retention [26,39-43], and that clients who enter treatment under legal pressures show comparable or better short-term treatment responses (e.g., reductions in substance use, criminal activity) to others in treatment [25,39,41,43-49]. These findings are typically interpreted as evidence of the effectiveness of legal pressure strategies. In addition, reviews of studies evaluating mandatory educational or therapeutic interventions for those convicted of impaired driving support their effectiveness in reducing impaired driving recidivism [50,51]. Finally, evaluation work conducted in drug treatment courts has largely concluded that these programs are successful in reducing drug use and criminal activity, at least for the duration of the program [52,53]; although this line of research has fallen under criticism for a number of conceptual and methodological reasons [14,54].

Pressures and mandates from employers have also met with mixed results, including longer retention [55] and a greater likelihood of program completion [56], as well as a lack of differences in retention [57] or substance-related outcomes [56,58] relative to others in treatment. To date, outcome evaluations of those who are mandated to participate in addiction treatment as a condition of receiving social assistance are very limited. Much of the work that has been done has focused on the subpopulation of women and single mothers receiving public aid in the US. These studies generally report positive impacts of treatment on substance use [59-61] and employment outcomes, including job rates and earned wages [13,60-62]. However, these outcomes have not been gauged against equivalent comparison groups of non-mandated or untreated individuals. One study comparing those referred to treatment through the welfare system to self-referred clients found no difference in rates of treatment completion [63].

Studies of the general population have demonstrated the important role played by informal social networks in pressuring problem drinkers to change their behaviour and/or enter treatment [64-67]. Based on his work, Room has suggested that such pressures are sufficiently common that few people likely enter treatment without being spoken to or pressured by friends or family [68]. Accordingly, in clinical samples, family and friends are among the most common sources of pressures to enter alcohol and drug treatment [27,30-32]. Recognizing the power inherent in social networks, a number of intervention methods involving family and friends and aimed at encouraging or inducing a loved one to enter treatment have been developed and tested [69-73]. These vary in degree of confrontation and involvement of the target individual in negotiating the admission and treatment processes, and to the degree that they have been evaluated. Some evidence has been published suggesting that informal pressures, ranging from encouragements to organized interventions to prompt treatment entry, are associated with higher rates of treatment completion relative to self-referred clients [69,72], as well as a greater likelihood of regular attendance at 12-step meetings and methadone treatment [74]. Further study has linked social network pressures to higher rates of abstinence relative to problem drinkers who are not confronted by their friends and family [70], but lower rates of abstinence relative to others in treatment [75]. The methodological quality of these studies is variable, however. The impact of informal pressures, as they occur and interact with other sources of pressure to enter treatment, on outcomes during and following treatment is largely unknown.

In contrast to the substantial body of research evaluating social controls, studies incorporating client perceptions of coercion into evaluations of addiction treatment are rare. The most commonly used measure of perceived coercion in addiction treatment settings is the MacArthur Perceived Coercion Scale (MPCS), developed by researchers with the MacArthur Research Network to assess the perceptions of psychiatric inpatients of hospital admission processes [76]. The MPCS considers client evaluations of control and choice throughout the admission process at a global level (i.e., without reference to the source or agent of coercion). A newer measure, the Perceived Coercion Scale [18], allows for a source-specific assessment of coercion and has the advantage of being developed specifically for addiction treatment clients; although it has yet to be used in evaluations of treatment process and outcomes. A related line of research involves internal motivational processes, much of it being theory-driven studies of controlled and autonomous motivation for treatment. Empirically, perceived coercion, assessed with the MPCS, is associated with higher controlled motivation and lower autonomous motivation for treatment [21].

Autonomous motivation at admission has been associated with increased session attendance [22,77], longer retention [22,78], and lower rates of in-treatment drug use [77,79]. Controlled motivation has been associated with poorer session attendance among clients in methadone maintenance treatment [77], but longer retention in outpatient counselling and therapeutic community settings [22,78]. Among offenders mandated to residential treatment, perceived coercion was unrelated to either treatment completion or re-arrest in the 8 months following treatment [80]. In other studies incorporating post-discharge outcomes, admission levels of autonomous motivation have been associated with lower frequency of drinking 9 to 12 months after discharge from alcohol treatment [81], as well as increased smoking cessation rates up to 30 months following a brief intervention [82,83]. In addition, randomized trials have substantiated the efficacy of smoking cessation interventions based specifically on promoting and supporting the autonomous motivation of clients [84,85]. The context fostered in these trials, guided by SDT, involves providing choice and a meaningful rationale for any specific requests, minimizing pressures and controls, acknowledging clients' feelings, and offering personalized feedback [86,87]. This is similar in many ways to the context promoted by Motivational Interviewing (MI) techniques [88], which have also met with success in treating substance use problems [89-91] (see also [92-94] for more explicit comparisons between SDT and MI).

Although the number of studies is relatively small, there is nonetheless growing consensus of the importance, separate from the application of any social controls or pressures, of fostering and supporting autonomous motivation for achieving positive outcomes - a concept that is antithetical to coercion in treatment.

### Applying the findings: implications of research practices

At the outset of this review, it was suggested that much may be gained by a more thoughtful specification of the coercion construct, to better align it with actual experiences of the admission process. The implications of current definitions and research practices for interpretation and application of existing literature is now considered.

Typically, research in this area has employed simple group comparisons, in which clients with a specific, often chart-documented, form of pressure, referral or mandate are compared to others in treatment. However, what constitutes exposure to pressure, resulting in group membership, is not uniform across studies, involving highly variable levels of initial force and ongoing monitoring and surveillance. For instance, a workplace referral may indicate anything from informal suggestions by coworkers or supervisors that treatment be sought voluntarily, to formal conditions of treatment entry carrying the threat of job loss [95]. Once in treatment, urinalyses and ongoing assessments of work performance and treatment compliance may or may not be used as further leverage to promote behaviour change. The lack of consistency in specification of treatment conditions affects not only the mandated or pressured group, but also the comparison groups, which tend to be comprised of a heterogeneous mix of clients who may or may not be pressured or mandated to treatment by other sources. Apparent from the above review of empirical findings, this tendency toward narrow, mutually exclusive groupings is not likely reflective of client experiences of coercion or internal motivational orientations toward treatment. To the extent that all clients are affected by a balance of external and internal forces leading up to treatment entry, studies that use this kind of simple grouping strategy are limited in terms of what they can contribute to debates of effectiveness of coerced treatment.

Another concern with the use of non-equivalent comparison groups relates to illness trajectory and expected outcomes. Evidence suggests that legally mandated clients, in particular, are younger and at an earlier stage in their addiction and treatment careers than others in treatment [32,41,43,46,47,96-98]. This forms the basis of suggestions that coercion is an effective early case-finding strategy, bringing people into treatment before their addiction and other health and social problems become severe [99,100]. However, to the extent that these clients are systematically younger and less impaired by their substance use than those in the comparison group, their recovery process, trajectory, and prognosis may be different. Interpretations of differences in outcomes between groups, and the attributions of these differences to treatment, should be made cautiously.

The outcomes typically selected for evaluation have likewise limited what can be gained from this line of research. Evident in the review of empirical work presented here, retention figures heavily into evaluations of treatment under social controls and coercion. In a systematic review conducted 10 years ago, Wild identified retention has been the most commonly examined outcome in evaluations of compulsory treatment [5]. A focus on retention as a measure of outcome reveals implicit assumptions that treatment is both effective and that more is better than less [101]. To be sure, retention is a consistent predictor of positive outcomes across a variety of modalities [102-105]. However, at least for alcohol, it is also the case that brief treatment interventions are among the most effective [106], particularly for those with less severe impairment [107,108]. It is further questionable whether formal treatment always serves the best interests of the individual. Estimates suggest that between 7% to 15% of those who participate in addiction counselling programs show deterioration in their substance use and psychosocial well-being during or shortly after treatment [109]. Finally, it is also clear from population studies that the majority of individuals who experience substance-related problems recover without participating in a formal treatment program [110,111], highlighting that it is not a necessary component of the recovery process.

The meaning of retention in mandated and coerced treatment may be particularly limited. At the same time as being associated with better session attendance and longer retention, legal pressures have been linked with poorer cognitive engagement in treatment, described in terms of commitment to the treatment process and development of the therapeutic alliance [112]. Descriptions of clients "going through the motions" of treatment without actively engaging or participating in the therapeutic process have been documented as common incidents of non-compliance in both adults and adolescents mandated to treatment [113,114]. These findings highlight that, while physical presence in treatment may form part of client engagement, it does not guarantee meaningful participation [114,115]. To the extent that session attendance is already compulsory, retention-based measures may be particularly poor proxies for the internalization of treatment content and behaviour change [116]. Here, as with coercion, the personal perceptions of clients with respect to involvement in the therapeutic process are of potentially greater value. The preference for retention-based outcome measures, and interpretation of treatment "effectiveness" based on these measures, raises ethical questions about the intended purpose of mandated and coerced treatment.

Overall, the long-term impacts of treatment under social controls and coercion are largely unknown. There is evidence that initially beneficial outcomes of legally mandated treatment do not persist after the threat of sanctions is lifted [95,117,118] - a finding that is consistent with SDT predictions on the impact of controlled motivation for treatment and recovery [34]. However, outcomes related to quality of life and economic, relational, and psychological well-being in the longer-term have yet to be evaluated. If the targeted outcomes of coerced treatment involve stable recovery from addiction and the alleviation of burden to public health and safety, rather than social control or punishment, then effectiveness has arguably not been adequately demonstrated to date.

### The way forward

The evidence and insights presented here suggest a number of avenues for future research, along with some specific recommendations for methodological approaches toward the study of coerced treatment. Namely, chart-documented measures, such as mandates and referral source, should be supplemented with broader assessments of perceived limitations to autonomy in decision-making around treatment entry. Because clients are often experiencing multiple pressures to seek treatment, there is potential value in assessing pressures or social control strategies across domains (i.e., including all of legal, other formal, and informal sources), and incorporating dimensions of strength or importance in the decision to seek treatment. Note that these approaches are inconsistent with the tendency toward classifying clients into mutually exclusive groups.

In addition, a continued focus on retention to the exclusion of other measures of process and outcome is not likely to produce additional information of value to debates of effectiveness of coercion. To the aim of understanding the treatment *process*, attendance-based measures should be supplemented with additional measures of cognitive engagement and involvement. To the aim of understanding treatment *outcomes*, attendance-based measures should be supplemented with broader measures of recovery, including indicators of substance use and related problems, economic and psychological well-being, criminal activity and others. Comprehensive models of treatment-assisted recovery, incorporating both in-treatment and post-treatment outcomes, are available in the published literature [119,120], and may provide guidance in this area.

More generally, research is needed into social contexts of recovery from addiction problems, including identification of salient elements of treatment and informal support networks that promote or hamper the recovery process. Theoretical work on self-determination suggests that coercion and autonomy play a central role in this process, with consequences for stabilized recovery in the long-term. This and other theoretical work on health behaviour change may provide guidance on the selection of appropriate measures and in outlining the mechanisms through which they influence each other and outcomes. External pressures to enter treatment, broadly defined and qualified in terms of their meaning to clients, as well as perceptions of coercion, development of the therapeutic alliance, and receipt of social supports both within and outside of treatment are all constructs consistent with an SDT framework toward evaluating recovery. Other work that takes a life-course approach toward the study of addiction and treatment careers may also offer guidance in this area [121]. By explicitly incorporating aspects of the social context, including social capital and critical external and internal events, a life-course approach to the study of coerced treatment may help to clarify the relationship between pressures and coercion, as well as the circumstances under which social control strategies are most likely to achieve the desired outcomes.

Reflection on the desired outcomes of treatment under social controls and coercion is needed not only to guide measurement selection in research studies, but also for responsible policy development and service delivery. This includes development of appropriate and ethical treatment interventions, monitoring and surveillance practices, and responses by treatment and other professionals (e.g., legal authorities, employers, and social service providers) to incidents of non-compliance or lapses during recovery. As an example, the abstinence orientation of treatment under legal controls is arguably not reflective of a chronic illness model of addiction, which calls for recognition of the role of relapse and the potential for multiple treatment episodes over the course of recovery [14,122,123]. Abstinence-based programs with punitive sanctions may not be suitable for all individuals with substance use problems. Those with severe and entrenched disorders may be at a higher risk of failing, thereby incurring additional punishment rather than treatment. More generally, an emphasis on the use of compulsory treatment to the benefit and protection of society over that of the individual may result in the imposition of treatment even when it is found to be ineffective, or over a longer period of time than is strictly necessary for treatment purposes [124,125]. It has been suggested that treatment provided in, or mediated by, the legal system may be driven less by client need than by local practices and policies for dealing with drug-using offenders [7]. Attention to these issues is needed to inform policy and practice guidelines around the use of social control strategies, and to ensure that practices align with the intended objectives of treatment.

## Conclusions

The concepts of self-determination and autonomy have not traditionally played a large role in studies of treatment-assisted recovery from addiction problems. Discussions of the role of coercion in addiction treatment have instead tended to centre on public health concerns of addiction, economic productivity, crime and infectious disease [39,124,126]. As a result, it remains largely unclear to what extent many of the commonly employed methods for getting people into treatment may be detrimental to the treatment process and, by extension, longer-term outcomes.

The ethical dilemma posed by coerced addiction treatment is complex from a public health perspective. Substance abuse poses real threats to public health and societal well-being, and this provides a strong impetus for government and other formal institutions to intervene in the lives of those with addiction problems. Ethical frameworks for the justification of public health intervention cite factors such as effectiveness and necessity of the measure in promoting and/or protecting the health of the public, and safe-guarding a balance between positive and negative effects of the intervention, as relevant concerns in the debate over whether to infringe upon individual autonomy and liberty [127,128]. Applied to the case of coerced addiction treatment, evidence would have to be brought to bear on whether the proposed course of treatment is likely to be successful in alleviating current harm and preventing future harm to the public that stems from the individual's use of substances and whether it is a necessary means to achieve these ends. Once demonstrated, it also needs to be considered whether the benefits outweigh any negative consequences resulting from the infringement of the individual's right to make their own decisions relating to treatment. It is not at all clear that past research has satisfied these conditions. Ethical arguments such as these do not prohibit the legitimacy of using social control strategies or restricting individual rights in the name of public health, but they do offer guidance to those charged with devising and implementing policies in this regard.

It remains to be demonstrated whether the exposure to treatment among coerced clients is ultimately beneficial or harmful in the long-run for the individual and for the public. The arguments presented in this review are not meant to belittle the negative effects of addiction, which is itself highly detrimental to psychological well-being, functioning, and development, nor is it meant to downplay the potential benefits of treatment. However, it is also relevant that formal treatment is only one option for overcoming addiction problems and, as noted earlier, many recover without it or with only brief supports from non-specialized professionals. These considerations are highly relevant in the current context of an increasingly widened net of addiction treatment, in which those who enter treatment under pressures appear to have less severe substance-related problems than those who are not pressured or mandated. The impact of coercion upon individual clients, treatment systems, and population health has not been adequately dealt with by addiction researchers to date.

## Competing interests

The author declares that they have no competing interests.

## Authors' contributions

This work constitutes a portion of the author's doctoral dissertation, conducted at the Dalla Lana School of Public Health, University of Toronto. The author conceived of and conducted all aspects of the review.
